# Salerno and Medical Women

**Published:** 1918-01-05

**Authors:** 


### GLIMPSES BACKWARD.*

### I.—Salerno and Medical Women.

Wanderers in Southern Italy will remember the town of Salerno, not so much for any distinctive beauties of its own, coming as it usually does when the mind is already satiated by impressions of loveliness, but as the starting-point of the termination of that pilgrimage around the coast which includes in its course the wonders of Sorrento, of Capri, Amalfi, Posilippo, and Maggiore. It is in truth pleasant enough in a sleepy, provincial fashion. The inevitable Corso Garibaldi runs the length of its unfinished sea-front, whereon the town band plays twice weekly the hackneyed melodies of Verdi and Leoncavallo. Behind its more modern buildings rises the cathedral, the gleaming tiles of its dome forming a conspicuous landmark as one rounds the little cape that shelters the harbour. Within the curious may find some really fine examples of *cinquecento* work, and the magnificent bronze doors adorned in niello work have, if I remember rightly, the distinction of a star in Baedeker. Here the casual tourist pauses, and having decided whether he will pursue the winding road that leads up the hill to Vietri or endure the rigours of an excursion to the temples of Paestum, lunches at the abominable *table d'hôte* and departs rejoicing. Yet here flourished from the ninth century, or possibly earlier, down to the thirteenth, a school of medicine as famous as any in Europe.

### The Development of Medical Education.

There are certain features in the medical education of our own time that we are rather prone to think of as originating with ourselves and as being indices of that evolution of humanity and progress in the study of mankind which are even now culminating in our own era. It is interesting, then, to study just how these developments came about, and to find that many of our startling innovations and discoveries have been anticipated not once, but many times in the chequered story of science. The Medical School of Salerno, as was natural in the dark days in which it had its birth, was intimately connected with monastic tradition. There is much that is obscure in the origin of this, the first, Italian Medical School. As early as the ninth century Salerno was famous for its great physicians; the names of several of them have been handed down to us. Ragenifrid, whom by his name one may assume to have been a Lombard, was Private Physician to Prince Wyamar of Salerno in the year 900. From early in the tenth century physicians from Salerno were frequently brought to foreign Courts to become the medical attendants of rulers. Patients of the highest distinction from all over Europe began to flock to Salerno, and with them came the inevitable horde of quacks, magicians, and nostrum-vendors. Here, in the salubrious air of the Southern Mediterranean, foreign potentates, high Church dignitaries, scholars and students of all nationalities collected. In the tenth century, we learn, the famous Bishop Adalberon went there when ailing, although he found no cure for his ills. Abbot Desiderius, however, a great Benedictine scholar of the time, who afterwards became Pope Victor III., regained his health at Salerno under the care of the great Constantine Africanus.

### The Salerno Medical School.

The medical school at Salerno was intimately connected with, if not, indeed, actually supported by, the Government. As early as the year 1140 King Ruggiero (Roger) of the Two Sicilies promulgated the law: "Whoever from this time forth desires to practise medicine must present himself before our officials and judges and be subject to their decision. Anyone audacious enough to neglect this shall be punished by imprisonment and confiscation of goods. This decree has for its object the protection of the subjects of our kingdom from the dangers arising from the ignorance of practitioners." Exactly a century later the Emperor Frederick II. extended his famous law to the Kingdom of the Two Sicilies, in which he laid down in much detail an exact schedule of the studies to be followed by students of medicine. As a preliminary training three years had to be spent in the study of logic, by which is meant the grammar and philosophy which composed the ordinary undergraduate course. Then followed three years devoted to medicine, to which another year must be added if a man desired to undertake the practice of surgery, when he would receive his degree, with, however, the proviso that yet another year was to be passed in the practical study of medicine under an experienced physician. The law further regulated the payment to be received per visit, which amounted to the wage usually paid to a labourer per day. The poor, however, were to be attended free. A doctor was not to sell drugs, nor might he derive any profits out of the business of an apothecary. Thus does history anticipate itself.

### A Mass of Medical Literature.

The Salernian School produced a mass of medical literature much of which, upon the introduction of printing at the time of the Renaissance, found its way into general circulation. When their surgery came to be written down it gave abundant evidence of the thoroughness with which this department of medicine had been cultivated by the Salernian Faculty. We have the text-book of Roger with the commentary of Rolando, and then the so-called commentary of the Four Masters. The most important medical writing that comes to us from Salerno, in the sense, at least, in the work that has had most influence on succeeding generations, has been most frequently transcribed, most often translated and committed to memory by many generations of physicians, is the celebrated Salernian medical

* Many of the facts in this and ensuing articles have been derived from Dr. J. J. Walsh's books *Old-Time Makers of Medicine* and *The Popes and Science*; Fordham University Press, New York.

**GLIMPSES BACKWARD**—(*continued*).

poem on Hygiene. Its title in the original Latin was "Regimen Sanitatis Salernitanum," and probably it dates from the beginning of the twelfth century. When, a century or more later, it became the custom to call medical books after flowers it became the "Flos Medicinæ" of Salerno, as at Montpelier we have the "Lilium Medicinæ."

### The Bedside Manner.

Another very interesting contribution bears the quaint title "The Coming of a Physician to his Patient." or "An Instruction for the Physician Himself." Some of the instructions run as follows: "When the doctor enters the dwelling of his patient, he should not appear haughty, nor covetous, but should greet with kindly, modest demeanour those who are present, and then seating himself near the sick man accept the drink which is offered him, and praise in a few words the beauty of the neighbourhood, the situation of the house, and the well-known generosity of the family." Again, "The fingers should be kept on the pulse at least until the 100th beat in order to judge its kind and character; the friends standing round will be all the more impressed because of the delay, and the physician's words will be received with just that much more attention." We are apt to consider the bedside manner a purely modern invention! The knowledge of this school, however, was not confined to vague generalities, for we find long and detailed descriptions of the symptoms and treatment of such conditions as intermittent fever, pneumonia, psoriasis, and consumption. For the latter they recommended the giving up of a strenuous life, laying great stress upon a suitable and generous diet and the necessity for graduated exercise in the open air. The need for an equable temperature was insisted upon in this and other pulmonary conditions, and although an almost unheard-of innovation, they insisted upon the heating of the patient's room.

### Medical Education of Women.

Not the least interesting chapter in the annals of Salerno is to be found in the opportunities provided for the medical education of women, and a relegation to their charge of a whole department in the Medical School, that of women's diseases. This, in view of the fact that the school was founded by Benedictine monks, makes it all the more surprising. But we must remember the proximity of Salerno to the great Grecian colony of Poseidon, where, as we know from many inscriptions, women were admitted to the Faculty, if not upon equal terms, at least with considerable facilities for practice in their special branch. Galen, for instance, quotes certain prescriptions from women physicians, and the name of one Cleopatra is handed down as the author of a work on Cosmetics. In Roman times women seem to have been admitted into the profession in large numbers, and to have been called in consultation by their male colleagues. In the light of such a tradition the presence of women professors at Salerno is less to be wondered at. The first definite evidence with regard to it comes in the life of Trotula, who seems to have been the head of her department. Her reputation extended far beyond her native town, and even Italy itself, and in later centuries her name was used to dignify any form of treatment for women's diseases that was being exploited. One of her books bears the intriguing title "De Passionibus Mulierum," whilst her major work seems to have been upon Obstetrics. In this she deals *inter alia* with prolapse of the uterus and the prophylaxis of rupture of the perineum. She was but one of a number of women physicians whose names and works have come down to us. While as teachers they had charge of the department of women's diseases, their writings would seem to indicate that they studied all branches of medicine. In the archives of Naples there are preserved a number of licences in which women are accorded the privilege of practising medicine. Apparently these licences were without limitation.

### Salerno's Decline and Fall.

The reputation of Salerno did not decline until the middle of the fifteenth century, when the attempt of the Kings of Naples to create a great university in their city finally procured its downfall. But the tradition thus established gradually spread westward, and it is not surprising that a little later we find the school of Bologna instituting a similar women's department. After Bologna came Paris, and last of all the movement spread to the Teutonic countries. In our own days the conflict which procured women a legal right to practise medicine is still remembered. "There is nothing new under the sun," and it is instructive to note that, notwithstanding their reputation, the Dark Ages were more advanced than—shall we say?—the mid-Victorian era.

